# Potent anti‐myeloma activity of the TOPK inhibitor OTS514 in pre‐clinical models

**DOI:** 10.1002/cam4.2695

**Published:** 2019-11-12

**Authors:** Andrew T. Stefka, David Johnson, Shaun Rosebeck, Jae‐Hyun Park, Yusuke Nakamura, Andrzej J. Jakubowiak

**Affiliations:** ^1^ Department of Medicine University of Chicago Chicago IL USA

**Keywords:** apoptosis, lenalidomide, myeloma, OTS514, TOPK

## Abstract

Multiple myeloma (MM) continues to be considered incurable, necessitating new drug discovery. The mitotic kinase T‐LAK cell‐originated protein kinase/PDZ‐binding kinase (TOPK/PBK) is associated with proliferation of tumor cells, maintenance of cancer stem cells, and poor patient prognosis in many cancers. In this report, we demonstrate potent anti‐myeloma effects of the TOPK inhibitor OTS514 for the first time. OTS514 induces cell cycle arrest and apoptosis at nanomolar concentrations in a series of human myeloma cell lines (HMCL) and prevents outgrowth of a putative CD138^+^ stem cell population from MM patient‐derived peripheral blood mononuclear cells. In bone marrow cells from MM patients, OTS514 treatment exhibited preferential killing of the malignant CD138^+^ plasma cells compared with the CD138^−^ compartment. In an aggressive mouse xenograft model, OTS964 given orally at 100 mg/kg 5 days per week was well tolerated and reduced tumor size by 48%‐81% compared to control depending on the initial graft size. FOXO3 and its transcriptional targets *CDKN1A* (p21) and *CDKN1B* (p27) were elevated and apoptosis was induced with OTS514 treatment of HMCLs. TOPK inhibition also induced loss of FOXM1 and disrupted AKT, p38 MAPK, and NF‐κB signaling. The effects of OTS514 were independent of p53 mutation or deletion status. Combination treatment of HMCLs with OTS514 and lenalidomide produced synergistic effects, providing a rationale for the evaluation of TOPK inhibition in existing myeloma treatment regimens.

## Graphical abstract

Multiple myeloma (MM) is currently considered to be incurable. For the first time, we show that an inhibitor of T‐LAK cell‐originated protein kinase suppressed several MM‐supportive pathways and induced potent MM‐selective killing that synergized with a common component of current treatment regimens, making OTS514 a suitable candidate for targeting in a clinical setting.

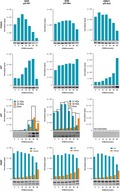

## 1 INTRODUCTION

Multiple myeloma (MM) represents a heterogeneous disease with various oncogenic mutations, chromosomal translocations, and copy number variations.^1^ MM progression follows a branching evolutionary path, generating subclones that shift in composition due to treatment and other selective pressures.^2^, ^3^ Major driver oncogenes include *IRF4* and *MYC*,^4^ and several of the common etiologic factors converge on aberrant activation of cyclin D genes and CDK4/6‐mediated cell cycle progression.^5^, ^6^, ^7^ Recent developments, particularly the use of proteasome inhibitors and immunomodulatory (IMiD) agents^8^, ^9^ with autologous stem cell transplantation,^10^ have dramatically improved overall survival, but relapse continues to occur necessitating the development of new therapeutic targets.^1^

T‐LAK cell‐originated protein kinase/PDZ‐binding kinase (TOPK/PBK) has been identified as a drug target due to its low expression in most normal tissues and high expression in various tumor types,^11^, ^12^ where high TOPK levels correlate with poor patient prognosis.^12^, ^13^, ^14^, ^15^, ^16^ Elevated TOPK expression is driven by MYC and E2F1 in high‐grade lymphomas^17^ and in FLT3‐mutated AML,^18^ but the mechanism of upregulation in other contexts is not well understood.

TOPK activity is associated with malignancy through multiple kinase pathways: it promotes tumor migration in lung cancer through PI3K/AKT signaling,^13^ proliferation of breast cancer cell lines through p38 MAPK,^19^ and chemoresistance in cervical cancer cells through NF‐κB signaling.^20^ TOPK also directly interacts with p53, inhibiting activation of cell cycle suppressor proteins and leading to aberrant tumor cell proliferation.^21^, ^22^

The TOPK inhibitors OTS514 and OTS964 have been successfully evaluated in several solid tumor types including lung,^12^, ^23^ ovarian,^16^ prostate,^15^ and kidney^24^ cancers. OTS514 has also shown promise in FLT3‐ITD‐mutated AML,^18^ but has not been evaluated in MM. In this report, we establish high‐level TOPK expression in MM and show for the first time that TOPK inhibition effectively kills MM cells. OTS514 induces cell cycle arrest and apoptosis, activating FOXO3 and CDK‐inhibitory proteins while disrupting pro‐survival kinase cascades. In addition, we show synergistic activity in combination with lenalidomide, which suggests a potential role of TOPK inhibition in current combination therapies.

## 2 MATERIALS AND METHODS

### 2.1 Cells, drugs, and viability assays

Human myeloma cell lines (HMCL) were obtained from ATCC (MM1.S [CRL‐9068], U266B1 [TIB‐196], NCI H929 [CRL‐9068], RPMI 8226 [CCL‐155]) or JCRB (KMS11 [1179]) and were confirmed mycoplasma‐negative and validated by 9‐marker Short Tandem Repeat profiling (IDEXX BioResearch). Other HMCLs were obtained from DSMZ (JJN3, LP‐1), ATCC (MM1.R), or RG Hawley (8226Dox40, KMS34, KMS34CFZ; George Washington University).^25^ Lysates from kidney cancer cell lines Caki‐2 and RCW were provided by Y. Nakamura. Cells were cultured in RPMI 1640 (Life Technologies, #72400) with 10% fetal calf serum and penicillin/streptomycin under 5% CO_2_. OTS514 and OTS964 were provided by OncoTherapy Science, Inc. Lenalidomide and verapamil were purchased from Cayman Chemical. For viability assays, 2 × 10^4^ cells/well were cultured in 96‐well plates with increasing concentrations of test drug for 72 hours (minimum 6 wells per dose per cell line). MTT (thiazolyl blue tetrazolium bromide, Acros Organics) was then added and incubated at 37°C for up to 1.5 hours. Cells were lysed overnight, absorbance at 570 nm was read, and IC_50_ was calculated using the four‐parameter log (inhibition) vs response fit in Prism 7 (GraphPad Software). Multi‐agent curves were evaluated for pharmacological synergy by the Chou‐Talalay method with CalcuSyn Software (ComboSyn, Inc).^26^

### 2.2 Flow cytometry assays

Fresh bone marrow aspirate was collected from patients with MM. Ficoll‐separated mononuclear cells were plated at a concentration of 2.5 × 10^5^ cells/mL with increasing concentrations of OTS514 for 18 hours. Cells were harvested and stained using the Annexin‐V‐FITC Early Apoptosis Detection Kit (#6592, Cell Signaling Technology) according to the manufacturer's protocol with the addition of APC‐conjugated anti‐CD138 antibody (clone DL‐101, Biolegend). For cell cycle analysis, cultures were plated in serum‐free media for 8 hours, then washed into complete media with or without test drug. Cells were harvested at the indicated time points and fixed with ice‐cold 70% ethanol. Later, cells were washed into PBS with 40 µg/mL propidium iodide and 100 µg/mL RNAse (Life Technologies) for analysis of DNA content. Data were collected on LSR‐II and LSR‐Fortessa instruments at the University of Chicago Flow Cytometry Facility and analyzed with FlowJo 10.4 (FLOWJO, LLC) employing the Watson (pragmatic) model for cell cycle analysis.

### 2.3 Western blots

Cells were lysed in RIPA with HALT protease/phosphatase inhibitor and protein was quantified, where indicated, using the Pierce detergent‐compatible Bradford assay kit (1861281 & 23246, Thermo Fisher Scientific). SDS‐PAGE was run with pre‐cast 4%‐15% or 4%‐20% gradient gels (Bio‐Rad) followed by semidry transfer to PVDF membrane (Millipore). Primary antibodies against FOXM1 (5436), phospho‐FOXM1 (14655), AKT (4691), phospho‐AKT (4060), p38 (9212), phospho‐p38 (4551), IκBα (4814), phospho‐IκBα (9246), Ikaros/IKZF1 (5443), IRF4 (4964), p21 (2947), p27 (3686), FOXO3a (2497), PARP (9542), and horseradish peroxidase‐conjugated secondaries (anti‐mouse IgG 7076, anti‐rabbit IgG 7074) were purchased from Cell Signaling Technology. Other primary antibodies included anti‐TOPK (612170, BD Biosciences), anti‐p21 (MS‐891‐P0, Thermo Fisher Scientific), and anti‐GAPDH (sc‐32233, Santa Cruz Biotechnology). Unless otherwise indicated, detection was performed with HyGlo (Denville Scientific) substrate using radiographic film. Fluorescent detection was performed on Odyssey (LI‐COR) with 800CW goat anti‐rabbit secondary after total protein staining and imaging with REVERT total protein stain (926‐332211 & 926‐11016, LI‐COR). Protein normalization and target quantification were performed using LI‐COR Image Studio. Individual bands of target protein were normalized to total protein in the same lane according to vendor's protocol.

### 2.4 Peripheral blood mononuclear cell stimulation and CD138 selection

Fresh blood was collected from patients with MM in sodium‐heparin tubes and was Ficoll‐separated to obtain the mononuclear cell (MNC) fraction. 2.5 × 10^6^ cells were plated in 3 mL RPMI in six‐well plates. Wells were left untreated or were stimulated with 5 ng/mL (each) recombinant human IL‐3 and IL‐6 (R&D Systems, 203‐IL and 206‐IL, respectively) with or without 10 nM OTS514. Cells were harvested after 6 days and CD138^+^ cells were separated using the EasySep Human CD138‐Positive Selection Kit (Stemcell Technologies). CD138^+^ and CD138^−^ cells were enumerated by counting with trypan blue exclusion.

### 2.5 Microarray analysis

5 × 10^5^ H929 cells were treated with 0.015% DMSO, 15 nM OTS514, 15 µM lenalidomide (LEN), or 5 nM carfilzomib (CFZ) for 24 hours. Additionally, each active drug combination was performed (OTS514/LEN, OTS514/CFZ, OTS514/LEN/CFZ, and LEN/CFZ). RNA from three independent experiments (a total of 24 samples) was extracted with the Qiagen RNeasy mini kit and analyzed on two Human HT12v4 bead arrays (Illumina) at the University of Chicago functional genomics core facility. Gene Set Enrichment Analysis (GSEA) was performed on quantile‐normalized, background‐subtracted data (BeadStudio, Illumina) using hallmark gene sets from the Molecular Signatures Database v6.1.^27^, ^28^ Upstream regulator analysis was generated through the use of Ingenuity Pathway Analysis (Qiagen Inc, https://www.qiagenbio-informatics.com/products/ingenuity-pathway-analysis).^29^ The full dataset is available in the NCBI's Gene Expression Omnibus (https://www.ncbi.nlm.nih.gov/geo/) under accession http://www.ncbi.nlm.nih.gov/geo/query/acc.cgi?acc=GSE128251.

### 2.6 Xenograft model

Xenografts were established in 5‐ to 6‐week‐old female NSG mice (#005557, The Jackson Laboratory) by subcutaneous injection in opposite rear flanks with 1 × 10^6^ or 2 × 10^6^ cells of the HMCL H929 with matrigel (Corning Life Sciences). When tumors reached 100 mm^3^, 50 or 100 mg/kg OTS964 or vehicle were orally administered to mice (n = 6 per group) 5 times per week by gavage. Body weight and tumor volume were assessed three times per week for 2 weeks. After 14 days, 4/6 mice were sacrificed, and treatment was withheld from the remaining mice while weight monitoring continued.

### 2.7 Measurement of peroxidase activity

MM1.S cells were washed and re‐suspended in HBSS containing 100 µM amplex red (A12222, Life Technologies). This suspension was aliquoted into microcentrifuge tubes, where equal volumes of indicated drug or control (2× concentration) were subsequently added. Controls include amplex red with the maximal dose of DMSO either with or without cells. Suspensions were added to opaque white 96‐well plates (781665, BrandTech) with repeated mixing between additions to four replicate wells per experimental group. Final concentration was 1 × 10^5^ cells per well with 50 µM amplex red. Plates were briefly centrifuged and incubated at 37°C for approximately 90 minutes after which fluorescence was detected on a Synergy HT microplate reader (BioTek Instruments) with excitation filter 530/25 nm and detection filter 590/35 nm. Gain setting was consistent between experiments. The average of the cell‐free control group was used for background subtraction.

### 2.8 Statistical analysis

Datasets fitting parametric assumptions were tested with ANOVA followed by Tukey's test for multiple comparisons between all groups or Dunnett's test for comparisons to a control group. Data not fitting parametric assumptions were tested with Friedman's test and Dunn's multiple comparisons test. Representative data reflect experiments performed at least three times unless otherwise stated. Mean (bars and single points) and standard error of the mean (error bars) are plotted throughout.

### 2.9 Institutional approval

The University's Institutional Review Board (IRB) and Institutional Animal Care and Use Committee (IACUC) approved all work involving human subjects and animals, respectively. Informed consent was obtained for all human subject research.

## 3 RESULTS

**Figure 1:**
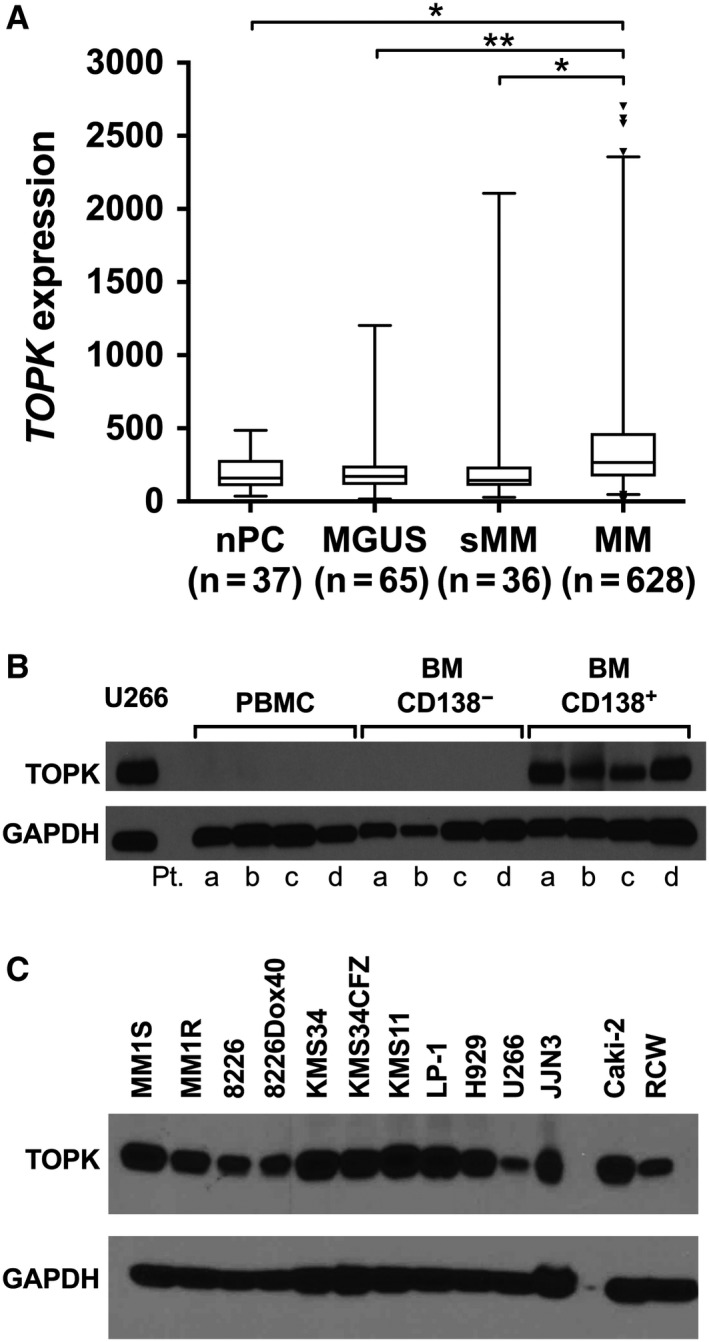
T‐LAK cell‐originated protein kinase (TOPK) expression is elevated in multiple myeloma (MM) plasma cells, but not in other cell types or precursor states. A, Analysis of published gene expression datasets^30^, ^31^, ^32^, ^33^ shows elevation of TOPK mRNA expression in plasma cells from patients with MM. Boxes represent median and 25th‐75th percentiles while whiskers depict 1st‐99th percentiles. B, Bone marrow aspirates from four patients with MM (labeled a‐d) were separated into CD138^+^ and CD138^−^ fractions by antibody/magnetic bead selection. Bone marrow cells and peripheral blood mononuclear cell from the same patients were analyzed by Western blotting for TOPK protein expression. U266 cell lysate as positive control. C, Western blot analysis of a panel of steady‐state human myeloma cell lines cultures shows robust TOPK protein expression in every line examined. Caki‐2 and RCW are kidney cancer lines used as positive controls. *P < .05, **P < .01, ANOVA with Tukey's multiple comparisons test

We first assessed TOPK as a potential therapeutic target by querying existing bone marrow plasma cell gene expression datasets.^30^, ^31^, ^32^, ^33^ *TOPK* mRNA expression was found to be elevated in plasma cells (PCs) from patients with MM compared with normal controls (nPC) (Figure 1A). The expression was disease‐stage dependent, as PCs from patients with the precursor states smoldering MM (sMM) and monoclonal gammopathy of undetermined significance (MGUS) exhibited significantly lower *TOPK* expression when compared to PCs from patients with myeloma requiring treatment. To further evaluate TOPK as a potential target in MM cells, fresh bone marrow aspirates (BMA) from patients with MM were separated into CD138^+^ and CD138^−^ fractions by magnetic selection and analyzed by Western blot along with PBMCs from the same patients (Figure 1B). TOPK protein was elevated in the malignant CD138^+^ PCs, but was not detected in PBMC or CD138^−^ bone marrow (BM) MNCs. We next assessed TOPK protein expression in a panel of HMCLs, which typically correspond to advanced disease. TOPK protein expression is easily detected in every cell line examined (Figure 1C).

**Figure 2:**
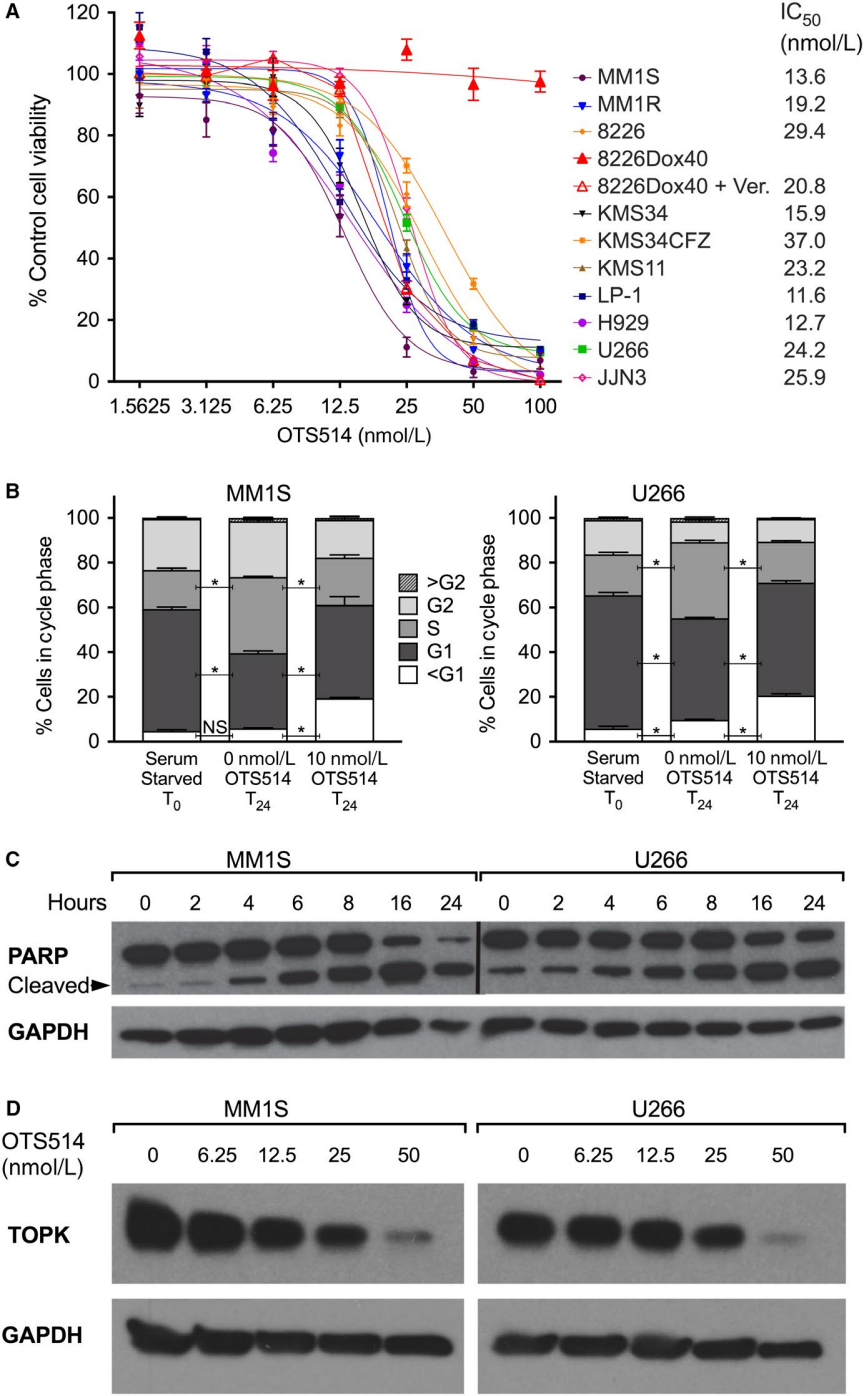
T‐LAK cell‐originated protein kinase (TOPK) inhibitor OTS514 is potently cytotoxic to human myeloma cell lines (HMCL), inducing loss of pro‐survival factors and rapid apoptosis. A, HMCLs were treated with increasing concentrations of OTS514 for 72 h and viability was assessed by MTT assay. The OTS514‐resistant cell line 8226 Dox40 was additionally cultured in the presence of 10 µM verapamil (+Ver), blocking ABCB1 activity and rescuing sensitivity. B, MM1.S and U266 cells were serum‐starved overnight to induce G1 arrest. Starvation was released and cells were left untreated or treated with 10 nM OTS514. After 24 h, DNA content was analyzed by flow cytometry with propidium iodide staining. C, HMCLs were treated with 100 nM OTS514 for 0‐24 h and PARP cleavage was detected by Western blot. Caspase‐mediated PARP cleavage was evident after 4 h. D, MM1.S and U266 were treated with OTS514 for 24 h and TOPK protein was analyzed by Western blot. TOPK inhibition caused loss of target protein in a dose‐dependent manner. NS, not significant. *P < .01 by two‐way ANOVA with Dunnett's multiple comparisons test

Having established that TOPK is upregulated in MM PCs, we used the TOPK‐selective kinase inhibitor OTS514^12^ to evaluate the potential of targeting TOPK in MM. We selected a broad panel of HMCL with significant diversity in primary IGH/IGL translocations and TP53 status to examine the cytotoxic potential of OTS514 using the MTT cell viability assay (Figure 2A). IC_50_ values ranged from 11.6 to 29.4 nM in parental cell lines, indicating a potent inhibitory effect. Only the RPMI 8226‐Dox40 cell line, which overexpresses the multi‐drug resistance transporter gene *ABCB1*, is resistant to killing by OTS514.^34^ We confirmed that this cell line's OTS514 resistance was conferred by ABCB1 overexpression by blocking the transporter with verapamil,^25^ which restored sensitivity. We next examined the effect of TOPK inhibition on the cell cycle (Figure 2B). MM1.S and U266 cells were serum‐starved to synchronize steady‐state cultures at the G1 phase, then given serum‐replete media with or without OTS514. After 24 hours, untreated cells had progressed into the S phase, whereas OTS514‐treated cells were arrested at G1 and showed an enriched population of cells with sub‐G1 DNA content suggestive of apoptotic DNA fragmentation. Activation of apoptosis by OTS514 was monitored over time by Western blot detection of PARP cleavage (Figure 2C), which was evident within 4 hours of high‐dose OTS514 treatment. In addition, TOPK protein level was decreased in a dose‐dependent manner (Figure 2D), consistent with previous reports in other types of cancer cells.^16^, ^23^, ^24^

**Figure 3:**
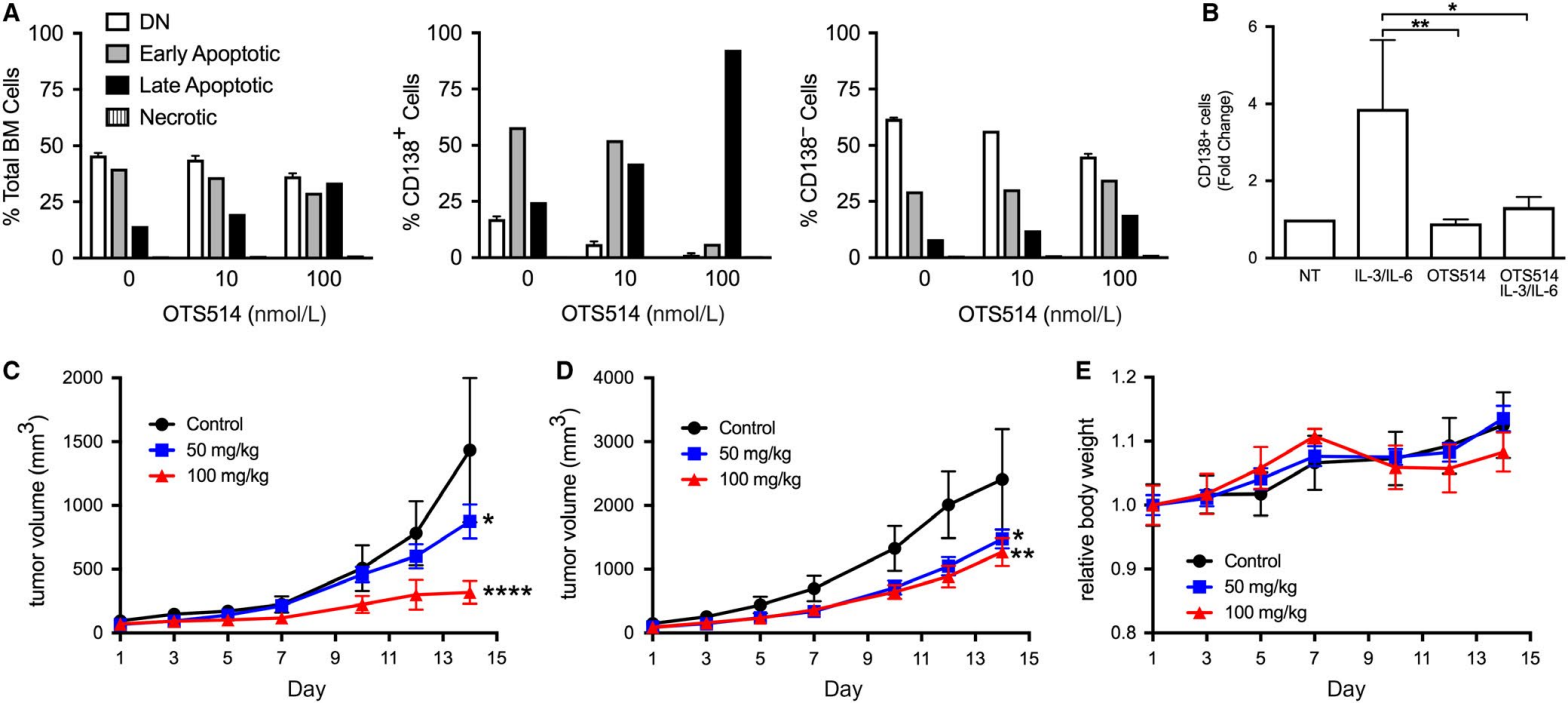
T‐LAK cell‐originated protein kinase (TOPK) inhibitor selectively kills CD138^+^ plasma cells (PCs) derived from bone marrow aspirates from patients with multiple myeloma (MM), prevents CD138^+^ outgrowth from peripheral blood mononuclear cell (PBMC), and is effective and well tolerated in a mouse xenograft model. A, Patient bone marrow mononuclear cells were plated with indicated concentrations of OTS514. Cultures were harvested and analyzed by flow cytometry after 18 h. Annexin V/PI staining shows effective induction of late apoptosis in the CD138^+^ PC fraction, with the CD138^−^ population remaining unaffected. B, PBMC derived from patients with MM were plated with/without 5 ng/mL IL‐3 and IL‐6 and with/without 10 nM OTS514. After 6 d, CD138^+^ cells were isolated by magnetic bead separation. IL‐3/IL‐6 treatment induced CD138 outgrowth from patient‐derived PBMC (n = 8); OTS514 treatment abrogated this effect. C‐E, Xenografts of H929 cells were established in NSG mice (NOD/SCID/IL2Rg), n = 6 mice per group. When tumor volume reached 100 mm^3^, mice began receiving OTS964 5 times per week. C, Tumor volume from injection of 1 × 10^6^ cells. D, Tumor volume from injection of 2 × 10^6^ cells. E, Body weight through the course of treatment. *P < .05, **P < .01, ****P < .0001, Friedman test with Dunn's multiple comparisons test (B) or repeated‐measures two‐way ANOVA with Dunnett's multiple comparisons test (compared to control) (C and D)

Having shown the effects of OTS514 in HMCLs, we sought to measure its effectiveness in samples from patients with MM using additional functional assays. BMA from patients with MM were collected as part of our normal tissue banking procedure. BM MNCs were isolated and treated with OTS514. After 18 hours, cells were harvested for analysis by flow cytometry (Figure 3A). CD138^−^ cells were largely unaffected by increasing concentrations of OTS514 while late apoptosis was induced in the CD138^+^ populations. To assess the effects of OTS514 on putative myeloma stem cell populations, PBMC derived from patients with MM were stimulated with IL‐3 and IL‐6, causing outgrowth of a CD138^+^ population. In the presence of OTS514, the emergence of CD138^+^ cells was suppressed (Figure 3B). We next used H929 cells to establish a xenograft model in NSG mice to test TOPK inhibition in an in vivo context. Once tumors were established, mice were gavaged with 50 or 100 mg/kg OTS964 (a dimethylated derivative of OTS514, for oral administration^12^) or vehicle 5 times per week. Growth of 1 × 10^6^ cells was inhibited by 81% at the highest dose given in a dose‐dependent manner (Figure 3C). Tumors arising from an injection of 2 × 10^6^ cells were inhibited by 48% at doses used (Figure 3D). OTS964 was well tolerated over the course of treatment as determined by assessment of body weight (Figure 3E).

**Figure 4:**
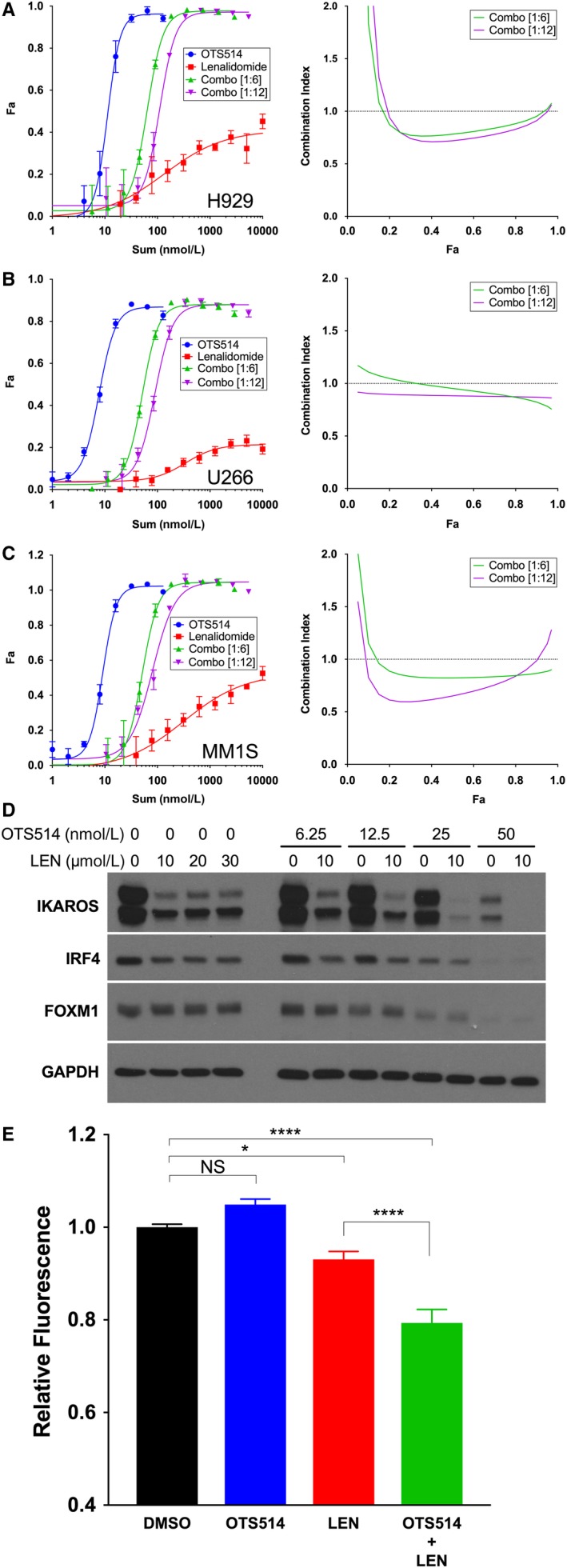
Synergistic activity of OTS514 with lenalidomide. A‐C, MTT assays were performed simultaneously with OTS514, lenalidomide, and constant‐ratio combinations (OTS514:lenalidomide = 1:6 and 1:12 molar equivalents). Effect level (*F*_a_) vs drug concentration and corresponding combination index for OTS514/lenalidomide combination are shown for (A) H929, (B) U266, and (C) MM1.S cells. Combination index <1 indicates pharmacological synergy. D, H929 cells were treated for 24 h with increasing concentrations of OTS514 with or without lenalidomide. Levels of IKAROS (IKZF1), IRF4, and FOXM1 were analyzed by Western blot. E, Amplex red peroxidase activity assay was performed using MM1.S cells with final concentrations of 20 µM lenalidomide, 100 nM OTS514, or the combination; DMSO equal to the maximal volume used to create drug dilutions was used as a control. NS, not significant, *P < .05, ****P < .0001, one‐way ANOVA with Tukey's multiple comparisons test

IMiD‐containing multi‐drug regimens are commonly used in myeloma treatment, so we next evaluated the interaction between OTS514 and the IMiD drug lenalidomide. Viability assays with fixed‐ratio combinations of the two drugs revealed pharmacological synergy in H929, U266, and MM1.S cell lines (Figure 4A‐C, respectively). We also detected additive loss of IKAROS/IKZF1 and the myeloma pro‐survival factors IRF4 and FOXM1 (Figure 4D). Since current hypotheses about the mechanism of action of IMiDs involve modulation of responses to oxidative stress,^35^ we assessed TOPK inhibition in an intracellular peroxidase activity assay (Figure 4E). The combination of OTS514 and lenalidomide led to an additive loss of antioxidative capacity. The proteasome inhibitor carfilzomib and the selective nuclear export inhibitor KPT‐330 were also evaluated in combination with OTS514 in HMCLs and exhibited no synergy by the Chou‐Talalay method (data not shown).

To interrogate downstream effects of TOPK inhibition, we treated H929 cells with OTS514 and performed a microarray. GSEA (Figure S1A) showed significant disenrichment in OTS514‐treated cells, particularly in gene sets related to cell cycle control (Figure S1B). Upstream regulator analysis confirmed that TOPK inhibition downregulated the FOXM1 pathway (Table S1).

**Figure 5:**
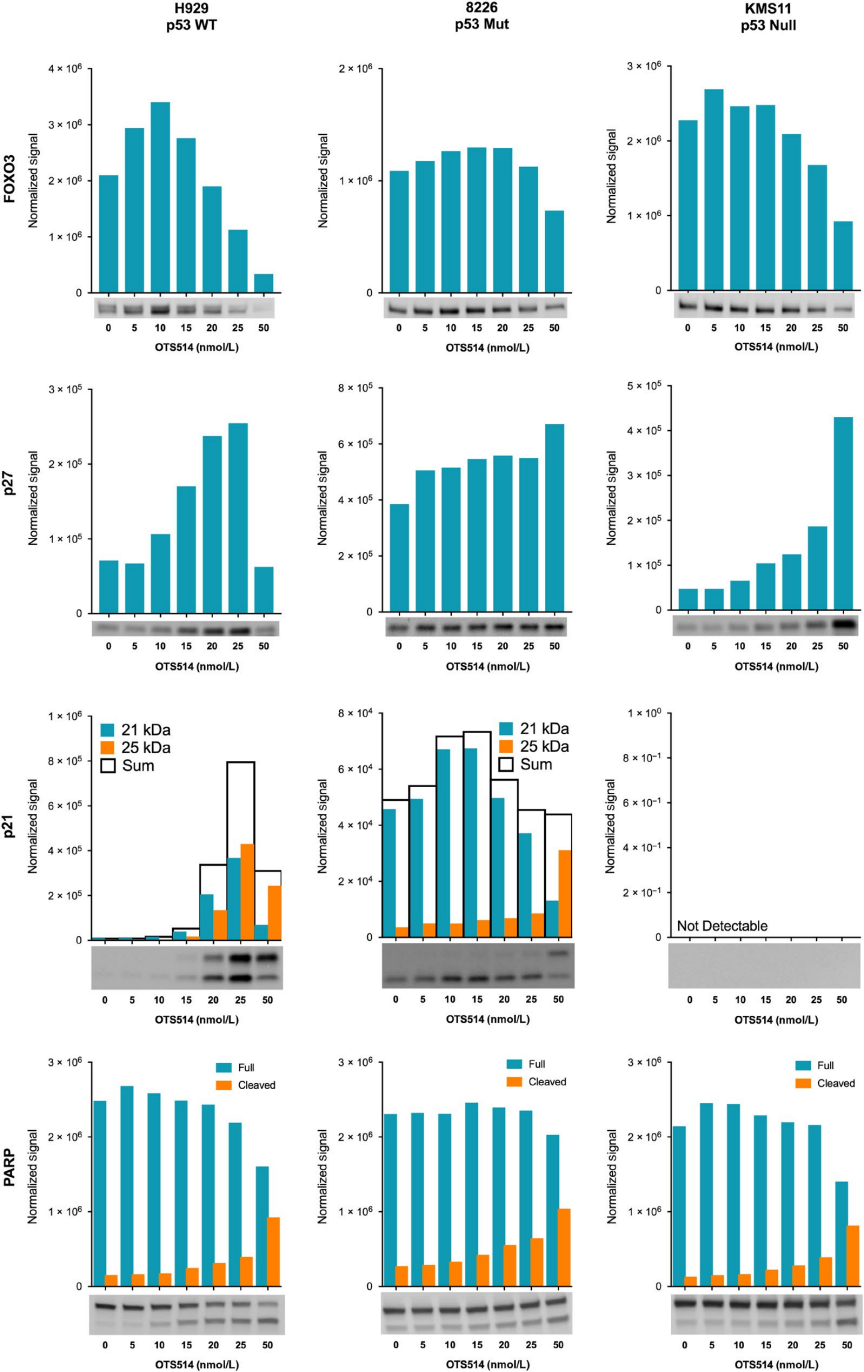
OTS514 activates a p21/p27 response irrespective of p53 status. H929, 8226, and KMS11 cells were treated with increasing concentrations of OTS514. After 24 h, Western blotting was performed with the LI‐COR near‐IR detection system. Loading was equalized for each cell line and further controlled by reversible fluorescent total protein staining and imaging. Fluorescent blot images are shown below bar graphs that indicate target signal normalized to total protein staining of the same lane

Having established a role for the cell cycle checkpoint machinery downstream of TOPK inhibition, we further examined the effects of OTS514 on the cyclin‐dependent kinase inhibitors p21 and p27. Using quantitative fluorescent Western blotting in a panel of cell lines chosen for their varied p53 status, we showed dose‐dependent elevation of p21 and p27 levels and PARP cleavage indicative of cell cycle arrest and apoptosis, respectively, in response to OTS514 treatment (Figure 5). In H929 and 8226 cells, p21 expression was elevated in a dose‐dependent manner accompanied by conversion to a 25 kDa isoform arising from a putative post‐translational modification (Figure 5 and Figure S2). OTS514‐induced total CDKN1A levels (21 and 25 kDa isoforms) begin to drop at the highest doses examined as apoptosis progresses. Although p21 protein was not detected in KMS11 cells by Western blot, dose‐dependent elevation of mRNA levels was evident by qPCR (data not shown). p27 was increased in all cell lines and the transcription factor FOXO3, which is involved in cell cycle regulation, was also elevated at moderate doses of inhibitor followed by a reduction at higher concentrations that coincided with PARP cleavage. This suggests an initial response of FOXO3 to OTS514 that precedes cell cycle perturbation and apoptosis.

**Figure 6:**
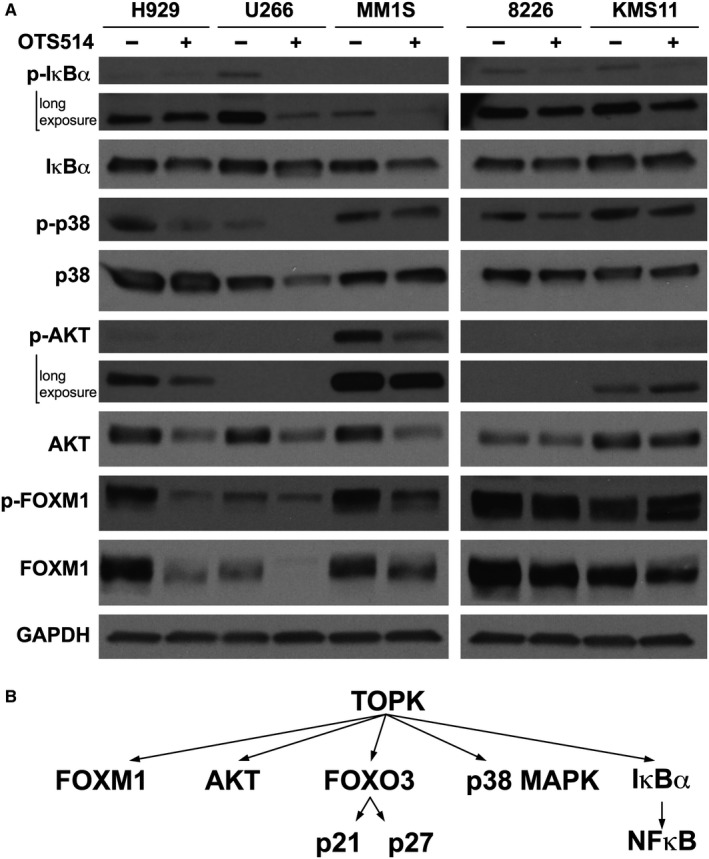
T‐LAK cell‐originated protein kinase (TOPK) inhibition affects multiple downstream kinase pathways. A, H929, U266, MM1.S, 8226, and KMS11 cells were treated with 15 nM OTS514 for 24 h. Loading was equalized for each cell line and Western blotting was performed using phospho‐specific antibodies for IκBα, p38, AKT, and FOXM1. Blots were then stripped and re‐probed to detect total levels of the same targets. Data shown are representative of two independent experiments. B, Schematic representation of kinase pathways affected by TOPK inhibition

Finally, we examined the ability of OTS514 to disrupt the activity of other cellular kinase pathways known to be targets of TOPK.^13^, ^19^, ^20^ FOXM1 phosphorylation or FOXM1 total protein levels were reduced upon OTS514 treatment in each of the five cell lines examined (Figure 6A). IκBα phosphorylation was reduced in all cell lines except for H929, in which the level of total IκBα protein was reduced. TOPK inhibition reduced phosphorylation of p38 MAPK in each cell line in our panel. Phospho‐AKT was not detected in U266 or 8226 cell lines, but was reduced in H929 and MM1.S cells. In p53‐ null KMS11 cells, p‐Akt was unexpectedly increased with OTS514 treatment, though p‐IκBα and p‐p38 are reduced as expected. Although there was heterogeneity in steady‐state kinase pathway status and in the subsequent response to TOPK inhibition between cell lines, suppression of oncogenic signal pathways was universal and resulted in cell death.

## 4 DISCUSSION

MM is a malignancy in which a common set of clinical manifestations is shared between patients, but not a common etiology. There is a complex genetic landscape in which a branching evolutionary path of progression leads to heterogeneity even within a single patient. This attribute necessitates treatment options that influence multiple pathways. TOPK, although its function remains to be fully clarified, has been shown to influence cellular proliferation, growth, migration, and stress responses important to MM, making it an ideal target. For the first time, we have shown that an inhibitor of TOPK suppressed several MM‐supportive pathways and induced potent MM‐selective killing that synergized with a common component of current treatment regimens, making TOPK a suitable candidate for targeting in a clinical setting.

Deletion of p53 in MM is more common in later stages of subclonal evolution, suggesting it may play a role in disease progression in affected patients.^36^ p53 mutation or deletion (including del [17p]) continues to be an indicator of high risk for relapsed/refractory disease, and post‐autologous stem cell transplant patients with p53 deletion have significantly worse survival.^37^ OTS514 treatment results in potent killing of 8 HMCLs carrying p53 mutations or deletions, including clear dose‐dependent p27 activation in p53‐null KMS11 cells. In p53‐mutant 8226 cells, the CDK inhibitors p21 and p27 were present at baseline (Figure 5), indicating that each mutational profile may require closer mechanistic study. These results indicate that the cytotoxic effect of OTS514 in myeloma cells does not require p53 sufficiency and, as such, OTS514 represents a possible treatment for this high‐risk patient population.

FOXM1, a member of the forkhead box family of proteins, is involved in the regulation of numerous pathways that promote cancer. Reflecting this importance, elevated FOXM1 has been observed in a subset of patients with newly diagnosed MM who exhibit significantly poorer overall survival after receiving front‐line therapeutics, such as proteasome inhibitors and IMiDs.^38^ FOXO3, another member of the forkhead box family, generally acts in opposition to FOXM1 and can decrease expression of FOXM1 while also interrupting its ability to act as a transcription factor.^39^, ^40^ By targeting a cancer's reliance on a deregulated balance of this FOXM1‐FOXO3 axis that is shifted in its favor, it can be driven from a supportive state to one that can lead to senescence and cell death. Our microarray data indicated FOXM1 pathway disruption, and we detected loss of phospho‐ and/or total FOXM1 protein levels with OTS514 treatment in every cell line examined, consistent with a previous report in kidney cancer cells.^24^ Additionally, levels of FOXO3 were seen to increase with higher concentrations of OTS514 followed by an eventual decrease when indications of PARP cleavage appeared. Interestingly, we show that OTS514 caused elevation of p27 levels in all cell lines examined and elevation of p21 in two of three cell lines. p27 and p21 are known targets of FOXO3 and their accumulation occurred at increasing concentrations of OTS514. FOXM1 is a known regulator of the cell cycle and acts in part through CDK4/6 as seen in one study where a CDK4/6 inhibitor induced loss of FOXM1 pro‐survival activity.^41^ p21 and p27 cause cell cycle arrest by binding and inhibiting cyclin‐CDK complexes, and progression through the G1/S checkpoint requires sequestration of p21/p27 by cyclin D‐CDK4/6.^42^ These differential changes in the expression levels of proteins involved with cell cycle progression comport with the G1 arrest seen with OTS514 treatment demonstrating the ability to target the FOXM1‐FOXO3 axis through TOPK inhibition.

The PI3K/AKT, MAPK, and NF‐κB pathways are implicated in the development of MM during which mutations and increased variability in the expression levels of oncogenes in these pathways leads to progression of MM to a more aggressive state.^43^, ^44^ In MM, where active phosphorylated AKT is present at baseline or is induced during treatment, the selective inhibition of AKT leads to perturbations of cell proliferation and promotes apoptosis.^45^ This suggests that in the two cell lines where levels of phosphorylated AKT decreased, the cytotoxic effects observed can partially be credited to this loss of active AKT. Activating mutations in the NF‐κB pathway are also known to drive MM.^44^ OTS514 treatment caused a marked decrease in the phosphorylated form of IκBα in four of the five HMCLs examined, indicating that TOPK inhibition also suppresses the canonical NF‐κB pathway and its MM‐supportive targets. MM promotes an osteolytic BM environment through a p38‐dependent mechanism.^46^ Upon inhibition of TOPK by OTS514, levels of phosphorylated p38 decreased in all cell lines examined, suggesting that targeting TOPK can reduce MM's ability to affect an oncogenic BM microenvironment. Collectively, these results demonstrate that OTS514 can disrupt multiple pathways in MM.

IMiD drugs such as lenalidomide are commonly used to treat MM, where they are often combined with proteasome inhibitors.^8^, ^9^ There are several hypothesized mechanisms of action for IMiDs, including CRBN‐mediated ubiquitin proteasome‐mediated degradation of IKZF1/IKZF3^47^ leading to loss of IRF4,^48^ among other pro‐survival factors. We show additive loss of IKZF1 and IRF4 when OTS514 and lenalidomide are given together. Sebastian et al propose an alternative mechanism of action involving inhibition of the antioxidative machinery necessary for MM cell survival.^35^ While OTS514 alone did not reduce antioxidative capacity as measured by the amplex red assay, we reconfirm that lenalidomide treatment reduces the peroxidase activity of HMCL and further show that the combination of lenalidomide with OTS514 resulted in greater loss of antioxidative capacity than did lenalidomide alone. It has been reported that TOPK, through its kinase activity, modulates the activity of the peroxidase Prx1 in melanoma cells by altering its conformation.^49^ A similar mechanism may be occurring in HMCL, though it is evident only after the intrinsically high antioxidative capacity of myeloma cells is overcome by lenalidomide treatment.^35^ All of the synergistic interactions reported here are intrinsic to the myeloma cells themselves, yet the clinical utility of IMiD agents is also attributed to modulation of the bone marrow immune microenvironment,^50^ where putative beneficial interactions will require further investigation.

## CONFLICT OF INTEREST

OncoTherapy Science, Inc: YN is a stockholder and scientific advisor. J.‐H. P. is an employee. The other authors declare no conflicts.
