# Supplementary material for: Potent anti‐myeloma activity of the TOPK inhibitor OTS514 in pre‐clinical models
Source: Cancer Med. 2019 Nov 12;9(1):324–34. doi: 10.1002/cam4.2695 (PMC6943155; doi:10.1002/cam4.2695)
Supplement: Supplementary file 1 [file CAM4-9-324-s001.pdf]

A

**Gene Set**	**Set Size**	**ES**	**NES**	**Nom. p-val**	**FDR q-val**	**FWER p-val**
HALLMARK_E2F_TARGETS	189	0.703	3.101	<0.001	<0.001	<0.001
HALLMARK_G2M_CHECKPOINT	190	0.642	2.850	<0.001	<0.001	<0.001
HALLMARK_MITOTIC_SPINDLE	197	0.532	2.394	<0.001	<0.001	<0.001
HALLMARK_GLYCOLYSIS	198	0.459	2.046	<0.001	<0.001	0.001
HALLMARK_FATTY_ACID_METABOLISM	156	0.437	1.868	<0.001	0.001	0.002
HALLMARK_MYC_TARGETS_V1	193	0.412	1.832	<0.001	<0.001	0.002
HALLMARK_HEME_METABOLISM	197	0.401	1.788	<0.001	0.002	0.008
HALLMARK_MTORC1_SIGNALING	196	0.397	1.763	<0.001	0.002	0.011

B

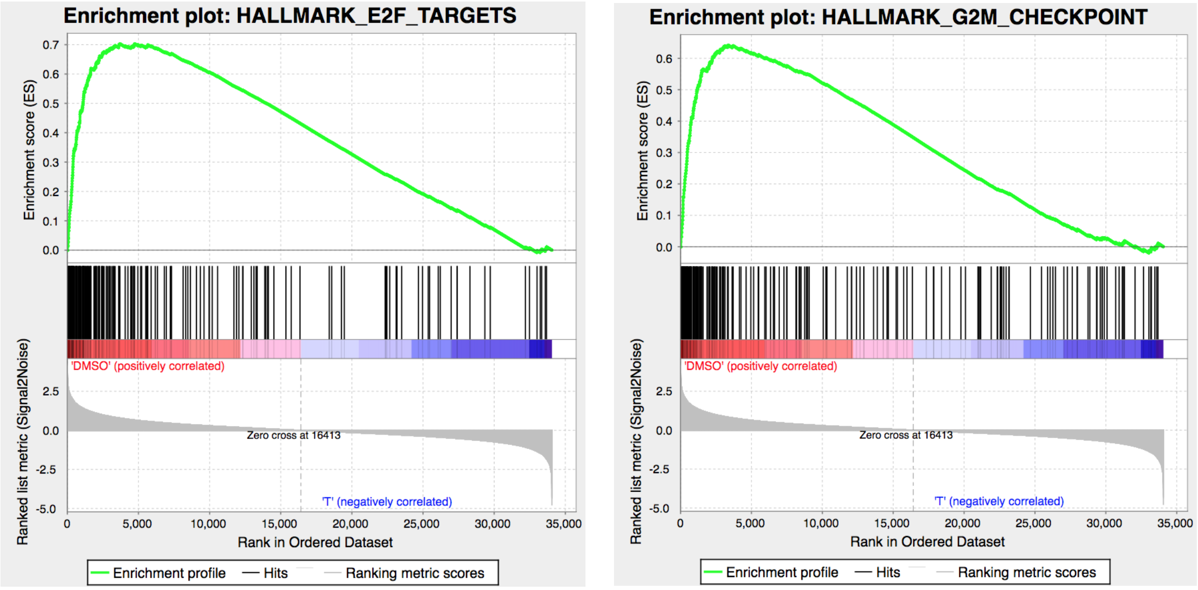

Supplementary Figure 1. Microarray analysis of OTS514 treatment. H929 cells were treated with 15 nM OTS514 or DMSO equivalent for 24 h. RNA from three independent experiments was analyzed by microarray. **A**. GSEA of hallmark gene sets. The gene sets most significantly disenriched by OTS514 treatment (enriched in DMSO controls) are presented. ES, enrichment score; NES, normalized enrichment score; FDR, false discovery rate; FWER, family-wise error rate. **B**. Full enrichment profiles for “E2F_TARGETS” (left) and “G2M_CHECKPOINT” (right).

Regulator	**FOXM1**		
z-score	**-5.143**		
Overlap p-value	**4.80E-15**		
Genes in dataset	Prediction (based on measurement direction)	Expr Fold Change	Findings
NEK2	Inhibited	-3.604	Upregulates
CDK2	Inhibited	-3.589	Upregulates
CENPE	Inhibited	-3.554	Upregulates
BIRC5	Inhibited	-3.432	Upregulates
CCNB1	Inhibited	-3.391	Upregulates
CENPA	Inhibited	-3.389	Upregulates
CCNE2	Inhibited	-3.297	Upregulates
AURKB	Inhibited	-3.035	Upregulates
CDKN3	Inhibited	-2.964	Upregulates
FOXM1	Inhibited	-2.699	Upregulates
CDK1	Inhibited	-2.661	Upregulates
PRC1	Inhibited	-2.584	Upregulates
CCNA2	Inhibited	-2.574	Upregulates
CDC25A	Inhibited	-2.566	Upregulates
GTSE1	Inhibited	-2.523	Upregulates
KIF20A	Inhibited	-2.514	Upregulates
CENPF	Inhibited	-2.392	Upregulates
CAV1	Inhibited	-2.323	Upregulates
LDHA	Inhibited	-2.227	Upregulates
CDC20	Inhibited	-2.036	Upregulates
CCNB2	Inhibited	-2.035	Upregulates
CDC25C	Inhibited	-2.012	Upregulates
CDCA8	Inhibited	-1.950	Upregulates
PLK1	Inhibited	-1.910	Upregulates
NBN	Inhibited	-1.898	Upregulates
PGK1	Inhibited	-1.882	Upregulates
MAPK8	Inhibited	-1.818	Upregulates
CKS1B	Inhibited	-1.805	Upregulates
CDC25B	Inhibited	-1.788	Upregulates
PCNA	Inhibited	-1.746	Upregulates
PLK4	Inhibited	-1.730	Upregulates
CDCA2	Inhibited	-1.668	Upregulates
CCND2	Inhibited	-1.659	Upregulates
BUB1B	Inhibited	-1.593	Upregulates
CDKN1A	Inhibited	2.878	Downregulates
STMN1	Affected	-2.413	Regulates
ATF2	Affected	1.958	Regulates
PTCH1	Activated	2.016	Upregulates
MYC	Activated	2.046	Upregulates
PRDX2	Activated	3.495	Upregulates

Supplementary Table 1.
Upstream Regulator Analysis result for FOXM1 after OTS514 treatment of H929 cells.
35 of 40 genes exhibit directionality consistent with inhibition of FOXM1.

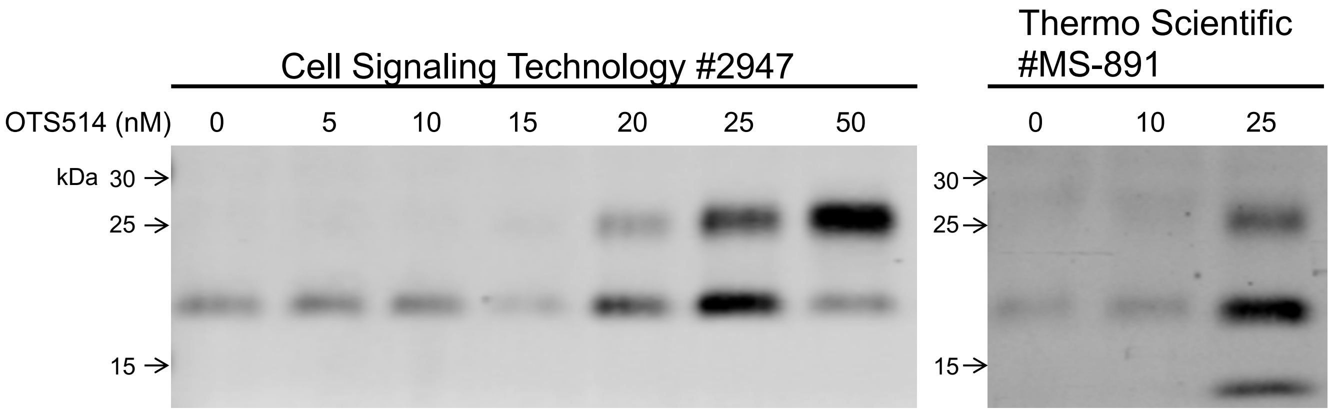

Supplementary Figure 2.
H929 Cells were treated with increasing concentrations of OTS514. Western blotting was performed with two different anti-p21 primary antibodies. With both antibodies, a larger species (~25kDa) occurs in a dose-dependent manner. The ~14kDa band observed with antibody MS-891 is indicative of apoptosis (Levkau *et al*. Cleavage of $p21^{Cip1/Waf1}$ and $p27^{Kip1}$ Mediates Apoptosis in Endothelial Cells through Activation of Cdk2: Role of a Caspase Cascade. *Molecular Cell* 1998; **1**: 553-563).
